# Bodily-tactile early intervention: a pilot study of the role of maternal touch and emotional availability in interactions between three children with visual impairment and additional disabilities and their mothers

**DOI:** 10.3389/fpsyg.2024.1439605

**Published:** 2024-07-29

**Authors:** Sini Peltokorpi, Saara Salo, Anne Nafstad, Paul Hart, Zeynep Biringen, Minna Laakso

**Affiliations:** ^1^Department of Psychology and Speech-Language Pathology, University of Turku, Turku, Finland; ^2^Pediatric Research Center, New Children's Hospital, University of Helsinki and Helsinki University Hospital, Helsinki, Finland; ^3^Faculty of Educational Sciences, University of Helsinki, Helsinki, Finland; ^4^Department of Combined Sensory Loss and Deafblindness, STATPED, Oslo, Norway; ^5^Sense Scotland, Glasgow, United Kingdom; ^6^Department of Human Development & Family Studies, Colorado State University, Fort Collins, CO, United States; ^7^Department of Psychology and Logopedics, University of Helsinki, Helsinki, Finland

**Keywords:** visual impairment, additional disabilities, early intervention, emotional availability, bodily-tactile modality, touch, early play routines, accessibility

## Abstract

**Background:**

Children with visual impairment and additional disabilities (VIAD) have difficulty accessing the visual information related to their parents’ facial expressions and gestures. Similarly, it may be hard for parents to detect their children’s subtle expressions. These challenges in accessibility may compromise emotional availability (EA) in parent–child interactions. The systematic use of the bodily-tactile modality for expressive and receptive communicative functions may function as a strategy to compensate for a child’s lack of vision. This multiple-case study explored the effects of a bodily-tactile early intervention for three mothers and their one-year-old children with VIAD.

**Methods:**

Video data from baseline, intervention, and follow-up sessions were analyzed using a bodily-tactile coding procedure and EA Scales.

**Results:**

During the intervention, all mothers began to use a more bodily-tactile modality in early play routines and in different communicative functions. They increased their use of anticipatory cues, noticing responses, and tactile signs. Moreover, the children were more emotionally available to their mothers during the intervention and follow-up compared to the baseline.

**Conclusion:**

The results indicated that, during a short intervention, mothers could adopt a systematic use of the bodily-tactile modality in interactions with their children with VIAD. The results also suggest that, when mothers increased flexibility in communication channels, it was positively linked to their children’s EA.

## 1 Introduction

Early parent–child interaction creates the basis for a child’s development. However, early interactions may be compromised if a child has a visual impairment (VI) and additional disabilities (AD), such as motor or intellectual disabilities or hearing impairment. AD is not a diagnostic category. It is a heterogeneous concept, as each child with VIAD can have different degrees and combinations of associated disabilities besides VI. Even normal hearing does not guarantee harmonious interactions between children with VIAD and their parents. Rowland (1984) found that early vocal interactions between children with VIAD and their mothers were characterized by a lack of reciprocity. Generally, children with VIAD can be less responsive and take fewer initiatives in interactions with their parents, which, in turn, can change their parents’ interaction style to be increasingly directive or intrusive (Grumi et al., 2021). The lack of responsiveness in children with VIAD may be due to their difficulties accessing visual information from their parents’ gazes, gestural expressions, and actions. Likewise, parents may not detect their children’s atypical gestural and bodily expressions (e.g., Fraiberg, 1971; Nafstad and Rødbroe, 2015). These challenges may hamper their emotional availability (EA) in early interactions (Sterkenburg et al., 2022).

EA refers to a safe, emotionally connected relationship between children and their caregivers (Biringen, 2008). This connection is expressed through eye contact, facial expressions, gestures, and words. An assessment using EA Scales focuses on the transactional relationship of the adult-child dyad. That is, adults’ and children’s behaviors are evaluated in relation to each other, rather than as separate behaviors (Biringen et al., 2022). EA has four scales for adults (sensitivity, structuring, non-hostility, and non-intrusiveness) and two scales for children (responsiveness and involvement). In typically and atypically developing children, EA is connected to their quality of attachment, empathy, emotion regulation, symbolic play, and social and language development (e.g., Pressman et al., 1999; Venuti et al., 2008; Biringen et al., 2014; Feniger-Schaal and Joels, 2018).

Children’s disabilities can compromise EA in parent–child interactions. However, research has shown that, on average, parents of children with disabilities have adequate EA, which means that their mean scores in the four adult dimensions are similar to those of parents of typically developing children (de Falco et al., 2009; Kubicek et al., 2013; Bentenuto et al., 2020; Shahar-Lahav et al., 2022). Nevertheless, parents of children with sensory impairments may experience difficulties in their emotional relationships with their children. Campbell and Johnston (2009) reported that sighted mothers of children with VI had some challenges in EA sensitivity and EA structuring. Similarly, Paradis and Koester (2015) found that hearing mothers of deaf children scored lower in EA sensitivity than hearing mothers of hearing infants. Children with disabilities show variability in their EA. Gul et al. (2016) found that a higher developmental age is associated with higher EA scores in children with autism spectrum disorders (ASD), other psychiatric disorders, and developmental delay. However, in the study by Bentenuto et al. (2020), the EA scores in children with ASD were predicted by symptom severity and not by their cognitive functioning. In children with cerebral palsy, lower EA scores have been found to be associated with hyperactivity and inattention (Barfoot et al., 2017).

EA between parents and their children with VIAD could be enhanced if the bodily-tactile modality (touch and movements) is used to improve the accessibility of interactions. Indeed, the research suggests that touch and EA have a positive link in interactions between mothers and their children with sensory impairments. Pipp-Siegel et al. (1998) found that in mothers of two-year-old children with hearing impairment or deafness, a lower EA score in nonhostility was related to decreased mutual touch in interactions, which suggests that touch is an important mode of communication with children with hearing impairments. Paradis and Koester (2015) also found a positive association between maternal touch and EA in interactions with deaf children. Moreover, they discovered that the EA dimensions had different correlations with the specific functions of maternal touch. Finally, Peltokorpi et al. (2020) discovered that the mother’s use of the tactile modality in imitating her child with congenital deafblindness (CDB) had a positive impact on their EA (mother and child dimensions).

Mothers with VI use touch naturally to compensate for their lack of vision in their interactions with their infants (Rattray and Zeedyk, 2005; Chiesa et al., 2015). Moreover, professionals have developed tactile strategies to support the communication and learning skills of children with VIAD (e.g., McLinden and McCall, 2002; Chen and Downing, 2006; Nicholas, 2010). This is based on the understanding that the same communicative functions can be conveyed by different modalities (Stern, 1985). Thus, tactile strategies differ from the typical use of social touch in interactions with children (*cf.* Cascio et al., 2019) and aim to transmit diverse social signals, such as the anticipation of actions (Goold and Hummell, 1993) or responsiveness in the bodily-tactile modality (Nafstad and Rødbroe, 2015). The tactile modality can also be used to convey cultural language through tactile sign language, which originates from visual sign language (Mesch and Raanes, 2023). In addition, early play routines can be modified to include a bodily-tactile frame. In this way, their content becomes accessible to children with VIAD and a resource for their participation through movements and gestures (Nafstad and Rødbroe, 2015).

Although tactile strategies were described in the literature decades ago, only a few papers have studied their benefits or the process of how sighted persons learn to use them. Therefore, this study investigated mothers’ use of the bodily-tactile modality by utilizing a bodily-tactile coding procedure. In an earlier study, Lindström (2019) found that care workers applied different tactile strategies in transforming information from visual sign language into bodily-tactile communication with a young man with a severe combined visual and hearing impairment. Moreover, Peltokorpi et al. (2020, 2023) discovered that an intervention increased mothers’ use of the bodily-tactile modality in interactions with their children with CDB and VIAD. The results suggested that the gestural expressions of the child with VIAD were based on his bodily-tactile play experiences. Similar findings have been documented in other studies (Ask Larsen, 2003; Brede and Souriau, 2016; Forsgren et al., 2018). However, otherwise competent parents of children with VIAD may not have knowledge of the compensatory use of the bodily-tactile modality and its potential for their children’s development. Thus, they may need early intervention to learn tactile strategies and how to access their children’s atypical expressions.

To date, only a few studies have explored the effects of early interventions for 0–2-year-old children with VIAD and their parents. Two studies used a video feedback intervention (Platje et al., 2018) and an interactive technology-based play mat, Barti-mat (Dyzel et al., 2023) to foster parents’ sensitivity. Other interventions utilized music therapy (Metell, 2015), daily routines (Chen et al., 2007), and early play routines (Rogow, 1982) to enhance interaction and communication between children with VIAD and their parents.

The utilization of play routines in early interventions (Rogow, 1982; see also Chen, 1996) is based on research showing that these games are optimal contexts for children to develop their communication skills through active participation (Ratner and Bruner, 1978; Fantasia et al., 2014; Nomikou et al., 2017). Parent-infant interaction games (e.g., nursery rhymes) are developmentally easier activities for children to join than games with objects (Trevarthen, 1980; Fantasia et al., 2014). Interaction games can be divided into “conventional games,” which include invariant conventional roles for the child and the adult (e.g., pat-a-cake) and “non-conventional games,” which do not have conventional roles (e.g., tickling; Camaioni and Laicardi, 1985). Parents embed their children’s actions in the games and respond to them as though their actions were intentionally produced (Rączaszek-Leonardi et al., 2013). This process enhances the children’s communication skills, and, gradually, their actions shape into gestures and words (Ratner and Bruner, 1978). Moreover, parents’ structuring of play has an impact on their children’s engagement (Nomikou et al., 2017; Fantasia et al., 2019). For instance, parents’ verbal and non-verbal actions related to anticipating upcoming actions help their children prepare for play sequences and participate in them (Nomikou et al., 2017).

A review of the research indicates a need for early interventions focused on the quality of parent–child interactions in families with children with VI (Grumi et al., 2021). However, at present, early intervention providers find their education inadequate to serve visually impaired young children (Ely et al., 2020), which necessitates evidence-based guidelines for professionals. The present study fills this gap in knowledge by investigating the effects of bodily-tactile early intervention on mothers and their children with VIAD. The intervention aimed to increase accessibility in early interactions between mothers and their children with VIAD through the bodily-tactile modality. It was designed based on wide literature on interventions for children with VI or VIAD (e.g., Fraiberg, 1971; Rogow, 1982), tactile strategies (e.g., McLinden and McCall, 2002; Chen and Downing, 2006), an intervention model for children with CDB (Nafstad and Rødbroe, 2015), and the mentalization-based parent–child intervention model called “Nurture and Play” (Salo et al., 2019).

Earlier intervention studies did not systematically guide parents to use the bodily-tactile modality in interactions with their children with VIAD. Moreover, none of them assessed EA in parent–child interactions. EA captures the dyadic emotional aspects of the parent–child relationship that underlie the development of secure attachment as well as the child’s emotional regulatory skills. Focusing on EA is important because children with intellectual disabilities are at an increased risk of attachment difficulties (Hamadi and Fletcher, 2021). Moreover, children with VIAD are at risk of developing emotional and behavioral problems (Alimovic, 2013), and there is a need to acquire more knowledge of the resources that may prevent problems from arising. Furthermore, it is important to gain more knowledge about EA in interactions between children with VIAD and their parents because there is a lack of research on the topic. Lastly, EA Scales could be a more appropriate method to use in assessing children with VIAD than other constructs, as they address both the adult and the child and contain guidelines for assessing children with disabilities.

### 1.1 Aims and hypotheses

The following research questions were addressed in this study: (a) How do mothers of children with VIAD use the bodily-tactile modality in early play routines and different communicative functions before, during, and after the intervention? (b) What is the quality of the emotional relationship between the mothers and their children with VIAD before, during, and after the intervention? We hypothesized that the mothers would increase their use of the bodily-tactile modality in interactions with their children with VIAD during the intervention and become more aware of their children’s bodily expressions, which, in turn, would elevate the scores on EA adult sensitivity and child responsiveness.

## 2 Methods

### 2.1 Participants

The participant children were searched with the following criteria: (a) maximum age of 2 years, (b) severe VI, (c) preverbal language skills (no more than 10 words or signs in use), and (d) Finnish as the language spoken at home. Staff members at university hospitals distributed an information letter about the study to the candidate families. The letter informed them that interactions between parents and their children with VIAD can be supported by guiding parents to use communication strategies that their children can understand. The letter also contained information about the place of the intervention (home or another environment, if preferred), the number and duration of the visits, the video-recording of the sessions, and the use of the videos as part of the guidance. The volunteer mothers contacted the first author by telephone, and the mother and father of each child gave written consent for their children’s participation. The study was approved by the ethics committee of the Helsinki and Uusimaa Hospital Districts, and it received a research permit from university hospitals in southern Finland.

Thea, Sara, and Alex were used as pseudonym names for the participant children. Their case histories were collected through medical records and a parental interview designed for this study. The interview consisted of questions related to the children’s early development, use of different senses, motor abilities, play, communication, emotional expressions, and family. All the children had cerebral/cortical visual impairment (CVI). There was no information available on the degrees of their VI. The additional disabilities of the children consisted of cerebral palsy and developmental delays. Sara received a diagnosis of intellectual disability during this study. All the children had epilepsy. However, the children’s hearing was reported to be normal. Only Sara had received speech therapy, which was delivered approximately once a month in her kindergarten. The mothers of Thea and Sara work as healthcare professionals, and Alex’s mother is a homemaker. The characteristics of the participating children are described below.

Thea (1.0 years old). The etiological causes of Thea’s disabilities were severe birth asphyxia and infection. She spent long periods in the hospital during her first 6 months. She then lived at home with fragile health and required nursing assistance. Thea had her first appointment with an ophthalmologist when she was nearly 2 months old and was diagnosed with CVI at the age of 3 months. She was able to detect moving toys with a strong contrast in color. Thea did not start reacting to sounds (e.g., rattles) until the age of 9–10 months. She could make eye contact for only a moment. However, she liked to make contact with other people through touch. Thea expressed herself through smiling, facial expressions, vocalization, and crying. She did not imitate her parents’ expressions. Her parents had used haptices (touch messages; Lahtinen, 2008) with Thea to anticipate actions (e.g., touching Thea’s chest before taking off her playsuit). Thea enjoyed early play routines in which her parents moved her hands and legs and provided her with strong sensory experiences. Thea’s mother expressed a wish to receive guidance on how to enhance reciprocal interactions with Thea and develop her use of touch messages.

Sara (1.9 years old). The etiological causes of Sara’s disabilities were prenatal hypoxic–ischemic encephalopathy and severe anemia as a newborn. She spent around 6 weeks in the hospital after birth. Sara had her first appointment with an ophthalmologist when she was nearly 1 month old and was diagnosed with CVI at the age of 3 months. Sara could see objects with distinct figures or bright colors and detected them best from a distance of 20–30 cm. She could make eye contact for short moments. She expressed herself through facial expressions, vocalizations, crying, and bodily expressions (e.g., kicking her feet or pulling her hand away). Sara smiled when she heard her parents’ voices or other interesting sounds and enjoyed being in her parents’ laps. She could sometimes vocally imitate her parents’ expressions if the parents first imitated her. Sara’s mother used toys and exercises from physiotherapy to play with her. She wished Sara could develop her expressive communication and that their interactions could become more reciprocal.

Alex (1.7 years old). The etiological cause of Alex’s disabilities was severe birth asphyxia. He was born preterm, spent approximately 1 month in the hospital after birth and needed periods of hospitalization afterwards. His parents received help with his care, and Alex spent weekends in a temporary care unit. Alex had his first appointment with an ophthalmologist when he had a corrected age of 4 months. The ophthalmologist’s examination suggested a brain-based VI. Alex liked being touched and close to others. He expressed himself through facial gestures, smiling, crying, laughing, and body movements. He did not imitate his parents’ expressions. Alex’s mother used stretching, chatting, and toys to play with Alex. It was not clear how much Alex could perceive visually; however, he had some functional vision. Alex’s mother wished Alex could communicate in some way and that he could have positive play experiences.

### 2.2 Design and procedure

**Figure 1:**
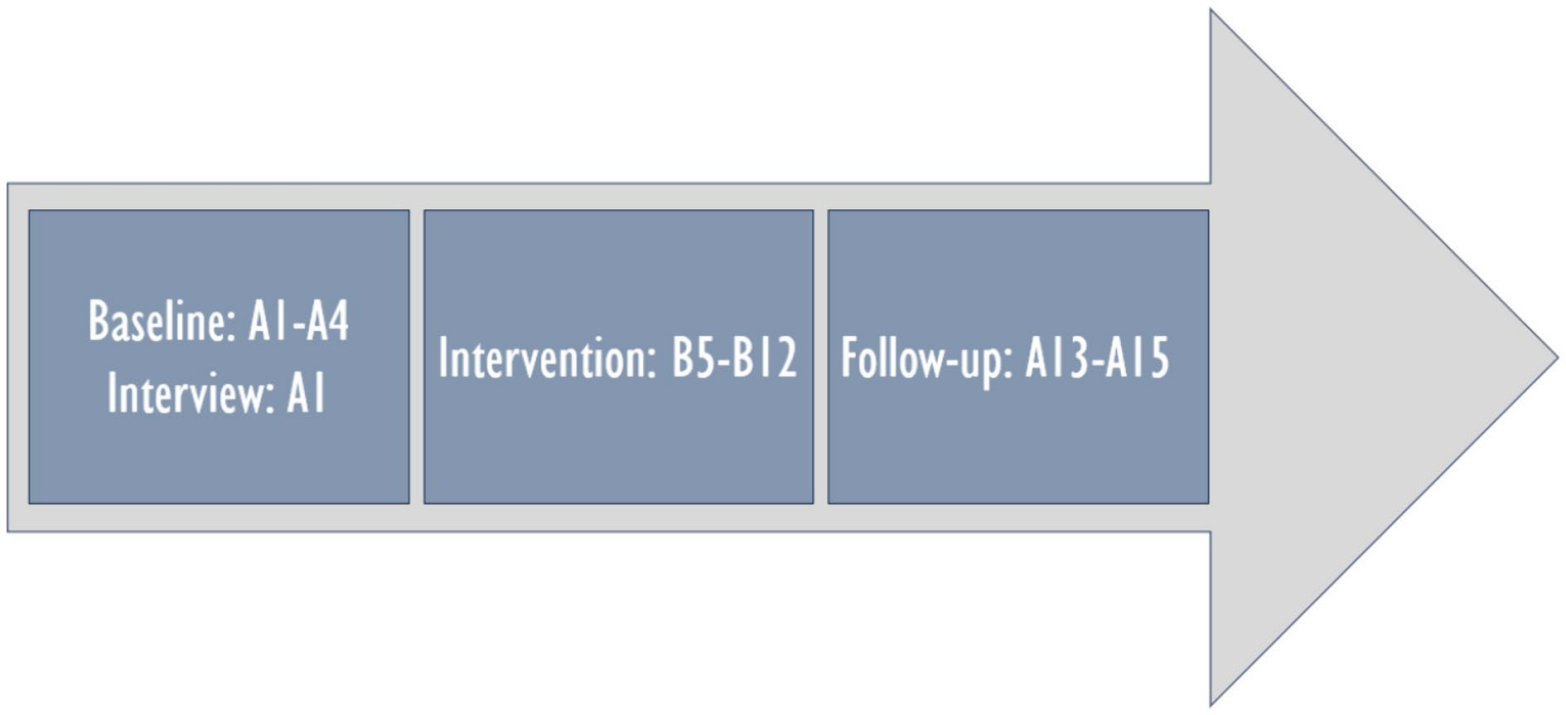
Data collection process.

This paper is a replication study of a pilot study (Peltokorpi et al., 2023), with modifications. Four baseline recordings instead of three were used to obtain a longer baseline period. The therapist met with each family 15 times in their homes. The parents were interviewed during the first meeting. Based on the parents’ decisions, only the mothers participated in the intervention with their children. Free play between the mother–child dyads was recorded with two video cameras in all the sessions. The optimal time of day for each child was chosen for the recordings. Two video recordings were made during each intervention session. The first recording included guided play with the therapist, the child, and the mother, and the latter included free play between the mother and her child. Before all the free play recordings, the mothers were asked to play with their children as they liked. During the recordings, Thea had nausea and difficulty breathing, Sara had frequent epileptic seizures, and Alex was medically very fragile and had frequent serious respiratory infections and breathing difficulties. After the data collection, each child received a toy gift card of 25 euros maximum. The process of data collection is illustrated in Figure 1. “A” refers to sessions without intervention and “B” to intervention sessions.

#### 2.2.1 Baseline

Four baseline recordings (A1–A4) were made weekly at home, with some exceptions. Due to Thea’s illness, there were 3 weeks between recordings A2–A3. For Alex, there were 2 weeks between recordings A1–A2 and A3–A4. During the baseline, the therapist did not inform the mothers about the content of the intervention to keep their interactions as natural as possible.

#### 2.2.2 Intervention

The intervention was founded on theories emphasizing the role of parent–child interactions in children’s development (e.g., Sameroff and Chandler, 1975; Vygotsky, 1978; Bronfenbrenner and Ceci, 1994). *The transactional model of development* highlights that children and their social environments have bidirectional effects on each other (Sameroff and MacKenzie, 2003). This model views the development of children as an outcome of the individual child and his or her experiences of social interactions with others over time (Sameroff and Chandler, 1975). Based on this theory, we focused the intervention on the mothers and aimed to make the mother–child interactions more accessible through the bodily-tactile modality. We expected that by increasing accessibility, there would be more reciprocity in their social encounters. First, we guided the mothers to use the bodily-tactile modality in early play routines and in different communicative functions. Second, we guided the mothers in detecting their children’s bodily expressions and responding to them through touch.

**Figure 2:**
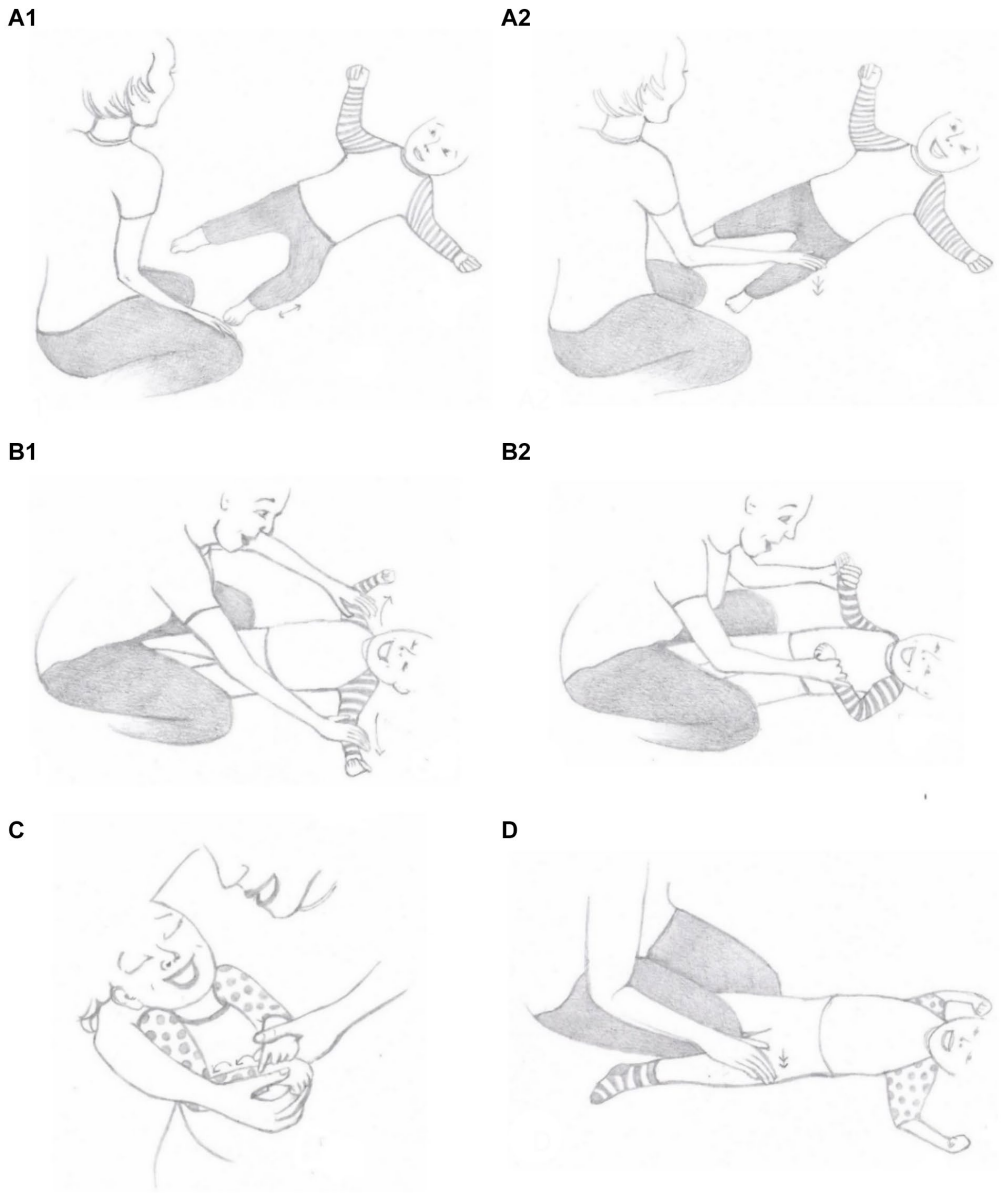
Illustrations of different communicative functions of touch: noticing **(A1,A2)**, anticipatory cues **(B1,B2)**, co-active signing (a modified sign) **(C)**, body sign **(D)**. Illustrations: Saara Koivula.

The eight intervention sessions (B5–B12) lasted a maximum of 90 min each. The average session lasted approximately 45–60 min. Thus, the total amount of intervention was approximately 480 min. The intervention sessions were recorded weekly at home, with some exceptions. Due to Alex’s illness, a one-week adaptation training course, holidays, and other family reasons, there were 2 weeks between recordings B7–B8, 3 weeks between recordings B8–B9, and 2 weeks between recordings B9–B10, B10–B11, and B11–B12. There were no exceptions in the recordings of Sara or Thea. After the last free play recordings (A4), the therapist informed the mothers of the content of the intervention and gave them their first instructions. The intervention was individualized for each mother–child dyad and built on their natural interaction patterns. That is, there were different emphases for the mothers regarding the themes of the intervention. The intervention was designed and implemented by the first author, a speech-language pathologist (MA) with a specialization in communication and CDB (MSc). After the intervention, the mothers were asked to give feedback using a feedback form and a video-recorded interview. The structure of the intervention sessions consisted of the following parts:

1. *Discussion and video feedback*. The therapist used discussion, video examples, and modeling to inform the mothers of how the bodily-tactile modality can be used in early social play routines and in different communicative functions. Moreover, the mothers were shown video clips demonstrating reciprocal interactions with their own children. The mothers received folders that included brief texts about the themes of the sessions and the lyrics of the songs, which aimed to help them memorize the information. They were also given an information sheet on useful books, articles, and webpages.
2. *Triadic play session*. During the play sessions, the therapist modeled the mothers’ bodily-tactile early play routines without toys. Early play routines without toys were utilized because they were considered developmentally easier activities for children to join than games with toys (Trevarthen, 1980). Moreover, the therapist modeled the different communicative functions of touch for the mothers, and the mothers repeated the modeled games and actions in interactions with their children after the therapist. The therapist informed the mothers that their children could use tactile contact instead of eye contact (Peltokorpi et al., 2023). She showed the mothers how to create slots for turn-taking through longer waiting times, which has been found to be important for increasing the participation of children with VIAD (Johnson and Parker, 2013). She guided the mothers to observe their children’s movements during the slots and notice them through the bodily-tactile modality (e.g., touching the child’s leg after his/her leg movement; Figures 2A1,A2). Noticing the children’s expressions through the sense of touch was used to inform them of their mothers’ attention and interest and to foster the children’s agency (Nafstad and Rødbroe, 2015). In addition, the mothers practiced how the bodily-tactile modality could be used for anticipating actions (e.g., touching the child’s hands before grasping them; Figures 2B1,B2). This aimed to help the children prepare for the actions (Goold and Hummell, 1993) and participate in them. Moreover, the therapist showed the mothers how imitation in the bodily-tactile modality can be used to enhance turn-taking (Hart, 2006; Peltokorpi et al., 2020). Furthermore, coactive signs (Figure 2C) and body signs (Figure 2D) were introduced as accessible forms of manual signs. In coactive signing, the adult makes the sign together with the child by guiding his or her hands in sign production (Chen and Downing, 2006). Body signs are manual signs that the adult makes on the child’s body (Lee and MacWilliam, 2008). While practicing these new tactile strategies, the mothers were also encouraged to continue verbal interactions with their children with VIAD as they normally did.
3. *Free play*. Free play between the mothers and their children was recorded during each intervention session. Lastly, the mothers were given suggestions on the functions of touch and bodily-tactile games that they could practice with their children the following week.

#### 2.2.3 Follow-up

The follow-up sessions were carried out 1, 5, and 9 weeks after the last intervention session, with one exception. Due to the coronavirus pandemic, Alex’s A15 recordings were made 24 weeks after the last intervention session. He stayed for about 3 months in the temporary care of children between recordings A14–A15.

### 2.3 Data analysis

Free play recordings from four baseline, three intervention, and three follow-up sessions were analyzed for each family. One recording from the beginning, middle, and end of the intervention, in which the child was most actively interacting with his or her mother, was used in the analysis. The selection was made by the first author based on a qualitative evaluation of the recordings. The length of the baseline, intervention, and follow-up recordings varied from 9 to 17 min for Thea, from 10 to 14 min for Sara, and from 10 to 11 min for Alex. The length of each recording was edited and rounded to the nearest full minute (e.g., 09:00 or 14:00) to compare the frequencies of the coded bodily-tactile phenomena per minute. Full recordings were used in the analysis with the EA Scales.

#### 2.3.1 Bodily-tactile coding procedure

ELAN (2021) (Version 6.2) software was used in the coding. The mothers’ bodily-tactile actions were coded in their contexts. That is, the previous and subsequent actions of the mother and child were noted when evaluating the relatedness between the actions. This was necessary to enable coding of the mothers’ anticipatory cues and touches related to noticing. Mutually exclusive categories were used in the coding (see the coding procedure in detail in the Appendix). However, if there were two bodily-tactile actions belonging to different categories appearing at the same time (e.g., a nonconventional play and a touch related to noticing), both actions were coded. This study used fewer categories in the analysis compared to the pilot study (Peltokorpi et al., 2023) due to the low frequency of some actions. Moreover, the categories of anticipating and noticing were divided into two categories. In addition, the definition of anticipatory cues was broadened to include movements (Goold and Hummell, 1993). Games with toys and mothers’ anticipatory cues related to toys were not coded because only interactional games without toys were utilized in the intervention. Furthermore, the coding of the touches and movements connected to the early play routines was developed further (see description below). The categories used in the coding were as follows:

##### 2.3.1.1 Early play routines

Camaioni and Laicardi’s (1985) coding procedure was applied to the coding. An early play routine was defined as an “interaction episode characterized by the repetition of specific behaviors or the presence of invariant conventional roles” (Camaioni and Laicardi, 1985). Early play routines were divided into nonconventional and conventional games.

###### 2.3.1.1.1 Nonconventional games

Camaioni and Laicardi (1985) coded a nonconventional play when at least one behavior made in the previous turn (e.g., motor or vocal component of a tickling game) was invariant in the same partner’s following turn, while other components of the previous turn could change. Camaioni and Laicardi (1985) included tactile, motor, visual and/or acoustic stimulation, and vocal or gestural imitation in the nonconventional games. In this study, a nonconventional game was coded when a child received repetitive bodily-tactile sensations with or without speech (e.g., the mother bounced the child in her lap). Moving, wiggling, massaging, or stretching the child’s arms or legs were coded only if the activities included sounds or melodies. Otherwise, it would have been difficult to evaluate which of the activities were social games. Tickling, swinging, and bouncing were coded only if there were at least two subsequent actions successively. Different stimulations were coded as one game if there were 5 seconds or less between the actions. Only the activities, including moving the child during the mother’s singing without conventional lyrics, were coded from the beginning until the end. Imitations were not coded as nonconventional games. This was done to avoid double coding.

###### 2.3.1.1.2 Conventional games

Camaioni and Laicardi (1985) coded conventional play in cases in which the play activity had invariant conventional roles (e.g., giving-taking or pat-a-cake). In this study, nursery rhymes and games with a systematic bodily-tactile structure, such as peek-a-boo or “Head, Shoulders, Knees, and Toes,” were coded as conventional games from the beginning until the end. The coding paused only if the child was unwell, causing the mother to stop playing and lift the child up. If the mothers played the same game twice successively, they were coded as one game if there were 5 s or less between the games. Play routines that consisted of tactile signing were not coded as conventional games to avoid double coding.

##### 2.3.1.2 Noticing

Touches related to noticing were defined as touches that the mothers made on their children’s bodies after noticing their bodily actions in the same locus (Figures 2A1,A2). The quality of touch related to noticing could vary (e.g., tapping or stroking). Three types of noticing behaviors were observed based on the data observations: (a) touches related to the mother’s responding to the child (e.g., the child moves her hand during the play, and the mother responds to it by touching or kissing the hand before continuing the play), (b) bodily-tactile imitation (e.g., the child rolls on the right side, and the mother imitates the movement with her body when the child’s legs are on the mother’s shoulders), and (c) contingent responses to the child’s reaching gestures in a form of action (e.g., the child reaches her hand toward the mother, and the mother responds to it by making a vibration sound on the child’s hand with her lips). If the mother made two or more subsequent touches related to noticing after the child’s movement, they were coded as one behavior if there were 5 s or less between them.

##### 2.3.1.3 Anticipatory cues

Anticipatory cues were defined as touches or movements that informed the child with VIAD about the following action (e.g., touching the child’s hands before grasping them; Figures 2B1,B2). Anticipatory cues are bodily-tactile variations of the *preliminaries* found in spoken conversations (Schegloff, 1980) and typical early interactions (e.g., Fantasia et al., 2019). Preliminaries project the following actions, are specific to them (e.g., pre-requests before requests), and involve the recipient’s acknowledgment (Schegloff, 1980). Thus, they help listeners orientate to the talk and respond at an appropriate time. Researchers of touch have referred to actions reminiscent of bodily-tactile anticipatory cues with terms such as touch-speech cues (Goold and Hummell, 1993) or haptices (Lahtinen, 2008). In this study, a touch or movement related to anticipation needed to correspond to the same place on the body as the following touch or movement to be coded. Typically, anticipatory cues occurred simultaneously with speech, but actions without speech were also included. Two anticipatory touches or movements were coded as one if there were 5 s or less between them. Tactile signs used for anticipating actions were not coded to avoid double coding.

##### 2.3.1.4 Tactile signs

Signs from Finnish Sign Language and self-created signs were coded as tactile signs if they were made as coactive signs (Figure 2C) or as body signs (Figure 2D). If the mother repeated a sign, the two subsequent signs were coded as one sign if there were 5 s or less between them. Moreover, whether a sign was made during a song or speech was registered. The signs during a song and speech were coded as separate signs, even if there were less than 5 s between them.

#### 2.3.2 The EA Scales

The EA Scales (4th edition) is an observational system that can be used to assess the emotional quality of parent*–*child interactions (Biringen, 2008). It has four subscales for the parent: *sensitivity* (the parent’s positive affect and her way of responding to the child), *structuring* (the parent’s ability to guide the play in a way that is received and responded to by her child), *nonhostility* (the parent does not show overt or covert signs of negativity), and *nonintrusiveness* (the parent’s ability to join the interaction without interfering with her child’s autonomy). The EA Scales have two subscales for the child: *responsiveness* (the child’s positive affect and willingness to respond to the parent) and *involvement* (the child’s initiatives in interaction). Each scale is rated from 1 to 7. The dimensions are rated as follows (Biringen, 2008). *Adult sensitivity*: 7 = highly sensitive, 5.5/6 = neutral sensitivity, 4 = inconsistently sensitive, 2.5/3 = somewhat insensitive, 1 = highly insensitive. *Adult structuring*: 7 = optimal structuring, 5.5/6 = moderately structuring, 4 = inconsistent structuring, 2.5/3 = somewhat unstructuring, 1 = non-optimal structuring. *Adult nonhostility*: 7 = nonhostile, 5.5/6 = generally nonhostile, 4 = covertly hostile, 2.5/3 = slightly overtly hostile, 1 = markedly and overtly hostile. *Adult nonintrusiveness*: 7 = nonintrusive but emotionally present/available, 5.5/6 = generally nonintrusive but sometimes benign forms of intrusiveness, 4 = “benign” intrusiveness, 2.5/3 = somewhat intrusive, 1 = intrusive. *Child responsiveness*: 7 = optimal in responsiveness, 5.5/6 = moderately optimal in responsiveness, 4 = complicated responsiveness, 2.5/3 = somewhat nonoptimal in responsiveness, 1 = clearly nonoptimal in responsiveness. *Child involvement*: 7 = optimal in involving behaviors, 5.5/6 = moderately optimal in involving behaviors, 4 = complicated involvement, 2.5/3 = somewhat nonoptimal in involving behaviors, 1 = clearly nonoptimal in involving behaviors.

The coder, who was trained and certified by the method developer, followed the guidelines for assessing children with disabilities in scoring the recordings (Biringen et al., 2005; Biringen, 2008). She was blind to the occasions of the recordings. The scoring was conducted per guidelines but with a flexible eye toward this specific population. (a) *Sensitivity*. There was no need to take special things into account, except that there was less focus on eye contact and more focus on voice, touch, general emotional tone, and approach to the child. (b) *Structuring*. Developmental information related to children’s motor and language development was considered. In particular, the use of both verbal and nonverbal guidance and the success of the attempts in structuring (as opposed to just mechanically repeating some play activity) were considered. (c) *Nonhostility*. No special flexibility in scoring was needed. (d) *Nonintrusiveness*. The children’s physical disabilities were considered, as many needed more stimulation and different-than-normal ways of holding them. (e) *Child responsiveness*. The level of communicative skills that each child had was taken into account. The children were given credit for any response or effort in responding (e.g., moving their hand or leg in response, turning their head toward the sound, or smiling). (f) *Child involvement.* Any attempt to make a connection or communicate was credited. Altogether, this special group of children was acknowledged and scored, keeping in reference low responsiveness and involvement in their group and not comparing them to typically developing children. Moreover, a consultation with the method trainer (Z. Biringen) was conducted to clarify the scoring of the data. As a result, the dimension of nonintrusiveness was rescored for each mother.

### 2.4 Reliability

Given that this is a pilot study, we used percentage agreement to indicate the reliability of the scoring because it is a commonly used measure to indicate interrater agreement (e.g., Kratochwill et al., 2010). The interrater reliability test related to the mothers’ use of the bodily-tactile modality in interaction was conducted with a second coder who had extensive experience working with children with sensory impairments and multiple disabilities and who was fluent in Finnish Sign Language. Altogether, 30% of the data from each mother*–*child dyad was recoded by randomly choosing one video from the baseline, intervention, and follow-up recordings (one video from each phase). Before coding, the second coder was trained to use the coding procedure with the non-analyzed data, and the information related to the coding procedure was rehearsed before each session of the reliability test. She was blinded to the phase of the intervention when coding the real data. The reliability of each coding category was calculated as the number of agreements divided by the number of agreements plus disagreements multiplied by 100. The interrater agreement on bodily-tactile early play routines was 88% for Thea’s mother, 83% for Sara’s mother, and 100% for Alex’s mother. The interrater agreement on noticing was 89% for Thea’s mother, 42% for Sara’s mother, and 96% for Alex’s mother. Because the interrater agreement on noticing for Sara’s mother was low, her results were not reported. The interrater agreement on anticipatory cues was 89% for Thea’s mother, 81% for Sara’s mother, and 83% for Alex’s mother. The interrater agreement was 100% on tactile signs for all mothers.

The interrater agreement of the EA analysis was made for 50% of the data from each family. The videos were randomly chosen; however, this was done in a way that allowed the videos from each phase (baseline, intervention, and follow-up) to be included in the reliability test. That is, two videos were randomly chosen from the baseline recordings, and a total of three videos were chosen from the intervention and follow-up recordings. However, it was ensured that at least one video from each phase was included in the test. The reliability test was conducted by another second coder who was a psychologist, trained to use the EA Scales and blinded to the phase of intervention. Before the reliability test, she practiced scoring with non-analyzed data in collaboration with the main scorer, following the guidelines for assessing EA in mother*–*child dyads with children with disabilities (Biringen et al., 2005; Biringen, 2008). She was also provided information on each child’s sensory functioning and development.

Due to the challenges in coding the data, one video (all EA dimensions) for Sara and three videos (all EA dimensions) for Alex were watched in consensus between the two coders. For Thea, one dimension from two videos was watched in consensus. All consensus scores were excluded from the reliability test. The final number of videos included for the reliability test was 50% for Thea (including videos from all phases of the study), 40% for Sara (including videos from all phases of the study), and 20% for Alex (including videos from the intervention and follow-up). However, there was no reliability test for the rescored EA nonintrusiveness because these were all consensus scores between the raters. When counting the maximum 1-point differences between the coders on each dimension, the percent agreement of the EA Scales was 91% for Thea and her mother, 90% for Sara and her mother, and 100% for Alex and his mother.

## 3 Results

All the mothers rated the intervention as very useful (5), using a scale from *not useful at all* (1) to *very useful* (5).

### 3.1 Mothers’ use of the bodily-tactile modality

#### 3.1.1 Early play routines

**Figure 3:**
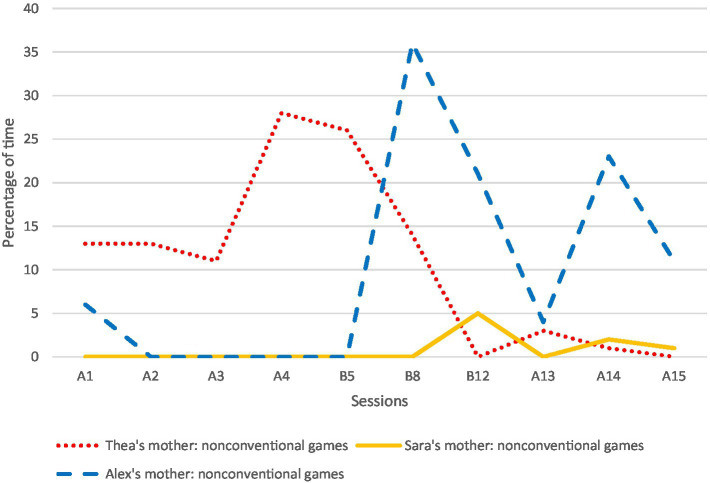
The mothers’ use of nonconventional games as a percentage of time per session. A1–A4 refer to baseline sessions (recordings were made weekly); B5–B12 refer to intervention sessions (recordings were made weekly); and A13–A15 refer to follow-up sessions (recordings were made 1, 5, and 9 weeks after the last intervention session). B5 refers to the session at the beginning of the intervention (B5 or B6), B8 to the session in the middle of the intervention (B8 or B9), and B12 to the session at the end of the intervention (B11 or B12).

**Figure 4:**
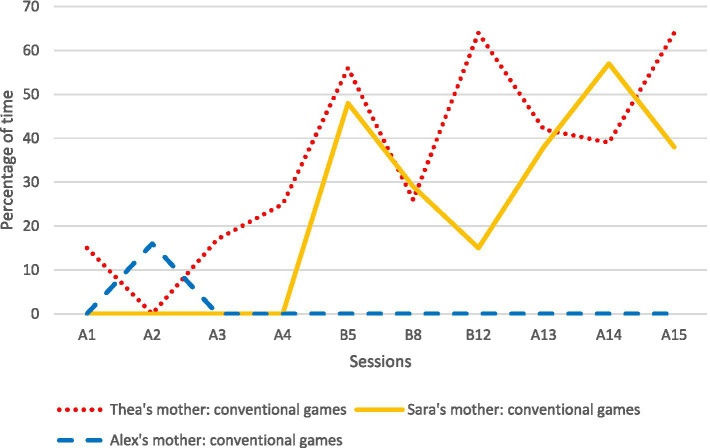
The mothers’ use of conventional games as a percentage of time per session. A1–A4 refer to baseline sessions (recordings were made weekly); B5–B12 refer to intervention sessions (recordings were made weekly); and A13–A15 refer to follow-up sessions (recordings were made 1, 5, and 9 weeks after the last intervention session). B5 refers to the session at the beginning of the intervention (B5 or B6), B8 to the session in the middle of the intervention (B8 or B9), and B12 to the session at the end of the intervention (B11 or B12).

The results related to the amount of time the mothers used bodily-tactile nonconventional and conventional games with their children during the sessions are presented in Figures 3, 4. Overall, the mothers used more bodily-tactile early play routines during the intervention and follow-up compared to the baseline. Thea’s mother played some nonconventional and conventional games with Thea during baseline. During the intervention and follow-up, she began to spend more time playing conventional games with Thea, while the amount of time used for nonconventional plays decreased. The play between Sara and her mother consisted mainly of toy play at baseline. During the intervention and follow-up recordings, she began to use conventional plays with Sara without toys. Similarly, Alex’s mother played with Alex mainly with toys in the baseline. During the intervention, she started using modeled and self-created nonconventional plays with Alex (e.g., swinging him in her lap and waiting for his responses).

All the mothers increased their ways of using the bodily-tactile modality in different communicative functions during the intervention, and this was also evident in the follow-up sessions. The results of the mothers’ use of noticing, anticipatory cues, and tactile signs are presented in Table 1.

**Table 1:** Frequencies of the mothers’ use of noticing, anticipatory cues, and tactile signs.

	**A1**	**A2**	**A3**	**A4**	**B5** ^**a**^	**B8** ^**a**^	**B12** ^**a**^	**A13**	**A14**	**A15**
**Thea’s mother**										
Noticing	4 (0,2)	0	3 (0,2)	3 (0,3)	20 (2,0)	37 (3,7)	10 (0,8)	17 (1,5)	17 (1,1)	14 (1,4)
Anticipatory cues	2 (0,1)	1 (0,1)	0	1 (0,1)	1 (0,1)	0	5 (0,4)	4 (0,4)	6 (0,4)	6 (0,6)
Tactile signs	0	0	0	0	0	0	17 (1,4)	16 (1,5)	17 (1,1)	0
**Sara’s mother**										
Noticing ^b^	–	–	–	–	–	–	–	–	–	–
Anticipatory cues	0	0	0	0	4 (0,4)	5 (0,5)	4 (0,4)	8 (0,7)	13 (1,3)	6 (0,5)
Tactile signs	0	0	0	0	0	8 (0,8)	15 (1,5)	5 (0,5)	6 (0,6)	9 (0,8)
**Alex’s mother**										
Noticing	0	0	0	3 (0,3)	2 (0,2)	11 (1,0)	14 (1,4)	33 (3,3)	11 (1,1)	9 (0,9)
Anticipatory cues	0	0	0	0	0	1 (0,1)	7 (0,7)	3 (0,3)	5 (0,5)	9 (0,9)
Tactile signs	0	0	0	0	0	9 (0,8)	0	0	0	0

The number in brackets shows the frequency of the phenomenon per minute. ^a^B5 refers to the session at the beginning of the intervention (B5 or B6), B8 to the session in the middle of the intervention (B8 or B9) and B12 to the session at the end of the intervention (B11 or B12). ^b^The results are not reported due to low reliability in coding.

#### 3.1.2 Noticing

At baseline, Thea’s mother used some contingent responses connected to Thea’s reaching gestures. When Thea reached her hand toward her mother’s mouth or face, her mother kissed Thea’s hand or made vibrations or other movements or sounds with her mouth against Thea’s hand. During the intervention, the mother’s noticing responses notably increased and changed qualitatively. Besides contingent responses, she began to respond to Thea’s bodily actions as turns by touching the part of body Thea had moved (e.g., leg) before she continued the game. She also treated Thea’s movements as initiatives or answers to her questions by touching the part of Thea’s body that she had moved and interpreting her actions verbally. Moreover, she noticed Thea’s actions by imitating her head movements. That is, when Thea turned her head, her mother imitated the movement with her body while Thea’s legs were on the mother’s shoulders.

Alex’s mother used touch to notice Alex’s expressions infrequently in the baseline. These occasions were when Alex moved his hand, and his mother (intuitively) touched his hand after the movement. During the intervention, this type of touch notably increased, as the mother began to use systematic noticing to respond to Alex’s movements. When Alex made a subtle movement with his hands, legs, head, upper body, or blinking eyes, his mother typically touched the part or close to the part of the body he had moved and verbally interpreted the action as an initiative. She imitated Alex’s leg movements on only one occasion.

#### 3.1.3 Anticipatory cues

Thea’s mother used anticipatory cues at times in the baseline. For instance, she moved Thea’s legs in the same way as in the subsequent song, when she asked Thea if she would like to play the game. Similarly, she touched Thea’s head before “Head, Shoulders, Knees and Toes” to inform her about this game. The frequency of the mother’s anticipatory touches and movements increased toward the end of the intervention and the follow-up. Most often, she used anticipatory movements connected to her questions. That is, she moved Thea’s legs or hands the same way as in the following song, when she asked Thea if she would like to play it.

Sara’s mother did not use anticipatory cues at baseline. During the intervention, she began to use anticipatory touches when she informed Sara about the following actions or asked her questions. Typically, she touched the part of the body (e.g., Sara’s hands) that she was about to touch or move next in the game. Sara’s mother also used movements associated with the games to anticipate them (e.g., she wiggled Sara’s legs when informing her verbally about the following verse, which included the same movement).

Alex’s mother did not use anticipatory cues at baseline. She began to use them during the intervention, and the frequency of anticipatory cues increased toward the end of the intervention and the follow-up. She used anticipatory cues in relation to her questions (e.g., touching Alex’s head when asking if he would like to play a swinging game) and when she informed Alex about the following actions (e.g., touching Alex’s hand before stretching it). She used remarkably more anticipatory touches than anticipatory movements when informing Alex about the following actions.

#### 3.1.4 Tactile signs

None of the mothers used tactile signs at baseline. Thea’s mother began to use co-active signing (Figure 2C) in “Itsy Bitsy Spider” at the end of the intervention. She made most of the signs during the songs. However, occasionally, she used signs during her questions when she addressed the “Itsy Bitsy Spider.” Sara’s mother began to use body signs (Figure 2D) and co-active signing with Sara in the songs “Itsy Bitsy Spider” and” A Little Dog Sings” during the intervention. She made the most of the signs during the songs. However, she also used some signs during her speech when she talked about the songs to Sara. Alex’s mother used the sign “DOG” many times in a self-created play during the intervention. She made all the signs during her speech, mostly by co-active signing.

### 3.2 EA between mothers and their children

The EA raw scores and means are reported in Table 2. No other statistical tests were used. During the baseline recordings, Thea’s mother’s mean EA scores for sensitivity were at the neutral level, her mean EA scores for structuring were at the moderately structuring level, and her mean EA scores for nonhostility were at the generally nonhostile level. Only her mean EA scores for nonintrusiveness were at a lower level, corresponding to “benign” intrusiveness. Her mean EA scores remained at the same levels during the intervention. However, there was a clearer change in Thea’s mean EA scores compared to her mother’s. During the intervention, Thea’s mean EA scores for responsiveness increased from the complicated responsiveness level to the moderately optimal level of responsiveness. Similarly, her mean EA scores for involvement increased from the complicated involvement level to the moderately optimal level in involving behaviors. Her mean EA scores for responsiveness remained at the moderately optimal level of responsiveness also during the follow-up.

**Table 2:** The means and ranges of EA for the children and their mothers before, during and after the bodily-tactile early intervention (Scale 1–7).

	**Baseline**	**Intervention**	**Follow-up**
**Thea’s mother**			
Sensitivity	5.8 (5.0–6.5)	6.2 (5.5–6.5)	5.8 (5.5–6.0)
Structuring	5.9 (5.5–6.5)	6.2 (6.0–6.5)	6.0 (6.0–6.0)
Nonhostility	6.3 (6.0–7.0)	6.7 (6.0–7.0)	7.0 (7.0–7.0)
Nonintrusiveness	4.6 (4.5–5.0)	5.0 (4.0–5.5)	5.0 (4.5–5.5)
Child responsiveness	5.4 (5.0–6.5)	6.3 (6.0–6.5)	6.0 (6.0–6.0)
Child involvement	4.3 (3.0–6.0)	5.7 (5.0–6.0)	5.3 (5.0–5.5)
**Sara’s mother**			
Sensitivity	4.8 (4.0–5.5)	5.7 (5.0–6.0)	5.3 (4.5–6.0)
Structuring	5.1 (5.0–5.5)	5.2 (5.0–5.5)	5.5 (5.5–5.5)
Nonhostility	5.8 (5.5–6.0)	6.2 (6.0–6.5)	6.0 (6.0–6.0)
Nonintrusiveness	4.4 (3.5–5.0)	5.5 (5.5–5.5)	5.3 (4.0–6.0)
Child responsiveness	4.1 (3.5–5.0)	4.7 (4.5–5.0)	5.2 (4.5–5.5)
Child involvement	3.5 (3.0–4.5)	4.0 (3.0–5.0)	4.5 (3.5–5.0)
**Alex’s mother**			
Sensitivity	5.1 (4.5–6.0)	5.5 (5.0–6.0)	5.7 (5.5–6.0)
Structuring	4.8 (4.5–5.0)	5.5 (5.5–5.5)	5.2 (5.0–5.5)
Nonhostility	6.3 (6.0–7.0)	7.0 (7.0–7.0)	7.0 (7.0–7.0)
Nonintrusiveness	4.9 (4.5–5.5)	5.3 (5.0–6.0)	5.8 (5.5–6.0)
Child responsiveness	4.8 (4.0–5.0)	5.8 (5.5–6.0)	5.3 (5.0–5.5)
Child involvement	3.8 (3.5–4.5)	5.3 (5.0–5.5)	4.8 (4.5–5.0)

At baseline, Sara’s mother’s mean EA scores for nonhostility were at the generally nonhostile level. Her mean EA scores for sensitivity corresponded to the inconsistently sensitive level, her mean EA scores for structuring corresponded to the inconsistent structuring level, and her mean EA scores for nonintrusiveness corresponded to the “benign” intrusiveness level. During the intervention, her mean EA scores for sensitivity increased from the inconsistent level to the neutral sensitivity level. Similarly, her mean EA scores for nonintrusiveness increased from the “benign” intrusiveness level to the generally nonintrusive level. However, during the follow-up, her mean EA scores for sensitivity and nonintrusiveness regressed back to the baseline level. At baseline, Sara’s mean EA scores for responsiveness corresponded to the complicated responsiveness level, and her mean EA scores for involvement corresponded to the somewhat nonoptimal level in involving behaviors. During the intervention, her mean EA scores increased in both dimensions, and she reached the level of complicated involvement in her involvement. Her mean EA scores for involvement remained at the level of complicated involvement also during the follow-up.

At baseline, the mean EA scores of Alex’s mother corresponded to the level of generally nonhostile in nonhostility, the level of inconsistently sensitive in sensitivity, the level of inconsistent structuring in structuring, and the level of “benign” intrusiveness in nonintrusiveness. During the intervention, her mean EA scores for sensitivity increased from the inconsistently sensitive level to the neutral sensitivity level. Likewise, her mean EA scores for structuring increased from the inconsistent structuring level to the moderately structuring level. Her mean scores for sensitivity remained at the neutral level also during the follow-up. At baseline, Alex’s mean EA scores corresponded to the complicated level for responsiveness and the somewhat nonoptimal level for involvement. During the intervention, his mean EA scores increased in both dimensions. His mean EA scores for responsiveness increased from the complicated responsiveness level to the moderately optimal level. His mean EA scores for involvement increased from the somewhat nonoptimal level to the complicated involvement level. His mean EA scores for involvement remained at the complicated level also during the follow-up.

## 4 Discussion

Our hypothesis was that the mothers would increase their use of the bodily-tactile modality in interactions with their children during intervention and become more aware of their children’s bodily expressions, which, in turn, would result in increases in EA adult sensitivity and EA child responsiveness. We found that the mothers increased their use of the bodily-tactile modality in interactions with their children with VIAD during the intervention. We also discovered that the mean EA values for adult sensitivity increased in all the mothers and changed from the inconsistent level to the level of neutral sensitivity in two of them. Similarly, the mean EA values for child responsiveness increased for all the children during the intervention and changed from the complicated responsiveness level to the moderately optimal level in two children.

### 4.1 Discussion of the main results

The results suggest that the mothers increased their use of the bodily-tactile modality in interactions with their children during the early intervention, which confirms the findings of previous studies (Peltokorpi et al., 2020, 2023). Moreover, our results suggest a positive link between the mothers’ increased use of the bodily-tactile modality and their children’s EA. That is, during the intervention, which targeted the mothers’ use of interactive and communicative touch and their ability to read and respond to their children’s bodily actions, their children had a higher EA. Our results correspond to the findings of Peltokorpi et al. (2020) and suggest a positive transactional effect of the intervention (Sameroff and MacKenzie, 2003). The findings provide new information about the bodily-tactile strategies parents could use to compensate for the lack of their child’s vision in interactions with their very young preverbal children with VIAD. These strategies may be helpful in creating a positive transactional process that leads to better developmental outcomes in children with VIAD and higher EA in interactions with their parents. Our results also indicate that the mothers found the bodily-tactile strategies helpful, as they rated the intervention very useful.

First, our findings indicate that the mothers and their children began to spend more time playing bodily-tactile play routines without toys during the intervention. The mothers of Thea and Sara increased their use of conventional games with their daughters, whereas Alex’s mother increased her use of nonconventional games with her son. The EA results suggest that both types of games were accessible to the children and appeared to create optimal contexts in their engagement, which is concordant with previous studies (Preisler, 1991; Ask Larsen, 2003). Moreover, in line with Trevarthen (1980), our findings suggest that interactional games without toys are developmentally easier activities for children to engage with than games with toys. Overall, the accessibility of bodily-tactile interactional games may increase the participation of children with VIAD. Thus, these games may be even more significant for this population than for typically developing children and applicable for a longer time.

Second, the mothers began to notice more of their children’s bodily actions through touches and movements during the intervention. This may be the result of a positive transactional process related to the intervention (Sameroff and MacKenzie, 2003). That is, the intervention could help mothers perceive more of their children’s movements and understand their potential as a means for participation. For example, at baseline, Thea’s mother interpreted only Thea’s reaching gesture as an initiative and responded to it by making sounds and movements with her mouth. During the intervention, she began to treat more of Thea’s hand and leg movements as initiatives. Those movements were not as clearly mother-directed as the reaching gesture, and their communicative potential could have been lost without the intervention. The same kinds of observations were made regarding Alex’s movements and his mother’s interpretations of his actions.

When the mothers noticed their children’s movements through a sense of touch, this could make their responsiveness more accessible to their children. Having better access to parents’ responsiveness is crucial because responsiveness plays a major role in the development of children’s social–emotional competences (Lang et al., 2017), intentional communication (Carpendale and Carpendale, 2010), and language (Laakso et al., 2010). Moreover, noticing may convey information about the mothers’ embodied mentalizing, which is the parents’ capacity to read their infants’ mental states (e.g., wishes) from their bodily actions and adjust their own bodily actions accordingly (Shai and Belsky, 2011). In this study, the mothers used three types of noticing responses to respond to their children with VIAD. It is possible that these bodily-tactile responses have different impacts on children’s participation in the same way as mothers’ diverse vocal responses (see Gros-Louis et al., 2014).

In the analysis, imitation was classified as one of the mothers’ noticing behaviors. This was also one of the themes of the intervention. That is, the mothers were encouraged to imitate their children’s movements in such a way that the children could perceive the imitations through the bodily-tactile modality. However, the mothers used imitation only occasionally to notice their children’s bodily expressions. This could be because parents typically imitate their children’s vocalizations and facial expressions more than their bodily actions (e.g., Papoušek and Papoušek, 1989). It is also possible that after detecting their children’s movements, the mothers found it more natural to touch the parts of their children’s bodies in which they observed a movement than to imitate the movements *per se*. As we did not code the children’s initiatives or responses to their mothers’ actions, the results do not indicate whether the children responded to their mothers’ imitations through re-imitations. However, our observations suggest that practicing imitation more systematically with the mothers might have been needed to create bodily-tactile imitative dialogues between them and their children. Hence, imitation through the sense of touch may require special attention and more time to be embedded in the interactional patterns of mothers and their children with VIAD or CDB (*cf.* Peltokorpi et al., 2020).

Third, the findings suggest that all the mothers increased their use of bodily-tactile anticipatory cues in interactions with their children with VIAD during the intervention, which is in line with the findings of Chen et al. (2007). This may be an important change in the mothers’ interactional behavior because previous studies have reported anticipatory cues useful for parents in understanding their children’s needs (Chen et al., 2007) and helpful for children with sensory disabilities in anticipating routines (Goold and Hummell, 1993; Chen et al., 2007), comprehending words, and developing communication through objects (Goold and Hummell, 1993). In general, the benefits of anticipatory cues could be based on their potential to make parents’ intentions perceivable for their children and contribute to a better sequential structuring of interactions (Fantasia et al., 2019). Thus, the use of anticipatory cues in interactions may create further positive transactions by helping children with VIAD participate in interactions (see Sameroff and MacKenzie, 2003).

Fourth, all the mothers began to use modified tactile signs from visual sign language with their children during the intervention. Similarly, the communication partners in the Lindström (2019) study were able to adapt visual signs into tactile forms and use them creatively with a young man with visual and hearing impairments. When tactile signs are used in early social play routines, children with VIAD gain access to cultural signs from early on, which may support their speech comprehension and sign acquisition. Body signs foster children’s receptive communication, whereas co-active signing also gives them a model for signing (Deuce and Rose, 2019). Thus, co-active signing could be more useful for children with VIAD in learning signs if their motor skills enable signing, and they do not experience tactile defensiveness (tactile hypersensitivity). In this study, we observed some tactile hypersensitivity in situations in which Sara’s mother used hand-over-hand guidance (see Chen and Downing, 2006) with Sara at baseline. In those moments, the mother took Sara’s hand and guided her to explore toys. Subsequently, Sara withdrew her hand from her mother’s grasp.

During the intervention, the mothers were encouraged to continue their typical style of verbal interactions with their children. As we did not analyze vocal aspects of interaction, the results do not indicate whether there were changes in the mothers’ speech or their children’s vocalizations during the intervention. However, our observations suggest that there were no clear changes in their verbal interactions. The children vocalized infrequently during all phases of the study, which allowed very limited possibilities for vocal reciprocity in the mother–child dyads. Thus, our results suggest that the intervention provided the mothers and their children with more resources for reciprocal exchanges through the bodily-tactile modality.

Finally, the results indicated that the mean EA values for EA sensitivity increased for all the mothers and changed from the inconsistent level to the neutral sensitivity level in two of them. Overall, the EA results indicated that all the mothers were well adapted to interactions with their children with VIAD at baseline. Their lower scores for nonintrusiveness can be understood from a transactional perspective. That is, the mothers’ leading role in interactions might have evolved from little engagement from their children with the typical means of interaction. During the intervention, the mothers’ mean EA scores were elevated for nonintrusiveness, and in Sara’s mother, the mean scores reached the generally nonintrusive level. These observations suggest that the mothers’ increased use of touch did not become intrusive. Campbell and Johnston (2009) also studied EA in the interactions of mothers and their one-year-old children with VI who did not have other disabilities. They found some challenges in the mothers’ EA sensitivity and structuring. Thus, it may be that the mothers who volunteered to participate in our study had more resources than the mothers of children with VI or VIAD in general. However, some families with infants with VIAD may also benefit from attachment-based interventions because severe VI in infants is a risk factor for higher parental depression, anxiety, and stress (Pur et al., 2023), and children with intellectual disabilities are at increased risk of attachment difficulties (Hamadi and Fletcher, 2021).

During the intervention, the mean EA scores for responsiveness improved in all the children. In two of them, the mean scores for responsiveness changed from the complicated responsiveness level to the moderately optimal level. Similarly, their mean EA scores for involvement were higher during the intervention. Thea’s mean scores for involvement increased from the complicated involvement level to the moderately optimal level in involving behaviors, and the mean EA scores of Sara and Alex increased from the somewhat nonoptimal level to the complicated involvement level. Sameroff and MacKenzie (2003) argue that these types of changes in children’s responsiveness and involvement during intervention programs aimed at parents provide evidence of transactional processes. Our results suggest that the main component of this process is increased accessibility. That is, during the intervention, the children with VIAD could perceive more responsiveness from their mothers to their bodily actions and access the shareable play routines that facilitated their participation. Our findings are also in line with other studies that found a positive association between maternal touch and EA in interactions with children with sensory impairments (Pipp-Siegel et al., 1998; Paradis and Koester, 2015). However, in our study, the bodily-tactile modality was used as a therapeutic strategy, which does not guarantee high EA. If adults concentrate more on therapeutic strategies than on children’s cues, this can lead to compromised EA (Barfoot et al., 2017). Thus, it is important that professionals consider EA in parent–child relationships when implementing interventions.

On average, the children’s mean scores for EA involvement corresponded to either the complicated or somewhat nonoptimal level of EA at baseline. That is, they took fewer initiatives than typically developing one-year-old children with VI (Campbell and Johnston, 2009) or a same-age child with VIAD (Peltokorpi et al., 2023). This may be due to the developmental stages of the participating children, which were below their chronological ages. Thus, the fact that the children differed in chronological age did not affect the coding or analysis. The children in previous studies used single words (Campbell and Johnston, 2009) or signs (Peltokorpi et al., 2023), whereas the children in this study expressed themselves with non-canonical vocalizations and movements. Indeed, Gul et al. (2016) found that higher developmental levels in children with autism spectrum disorder, other psychiatric disorders, or developmental delay were associated with higher child EA (responsiveness and involvement). Moreover, in this study, the type of play (with or without toys) and the children’s variable health conditions and alertness could have impacted their EA scores.

At follow-up, the mothers’ and children’s mean EA scores in different dimensions were either lower or higher than during the intervention. As the changes occurred in both directions, the interpretation of the results is challenging. However, as the mean EA scores decreased to a lower level in one or two dimensions for each mother–child dyad, it suggests that they may need longer interventions to maintain higher EA in interactions.

### 4.2 Discussion of the data and methods

The bodily-tactile coding procedure could capture the main changes in the mothers’ use of the bodily-tactile modality in interactions with their children. The low reliability related to noticing in Sara’s mother could be due to her personal and frequent use of the noticing type of touch, which was challenging to code. In future intervention studies, more emphasis could be placed on guiding mothers to make more articulated touches related to different communicative functions, which could make their communicative expressions more explicit to their children and facilitate coding.

The EA Scales were found to be useful for assessing the emotional relationship between the participant children with VIAD and their mothers. However, when the EA Scales are used for assessing EA in interactions with children with VIAD, it is important to have flexibility in scoring to avoid incorrect results and conclusions. Furthermore, despite the flexible scoring, the EA Scales may not detect micro-level changes in the interactions.

### 4.3 Limitations

This study has limitations that need to be considered when interpreting the results. First, this study had only three mother–child dyads as participants. Therefore, the results are suggestive and cannot be generalized to other children with VIAD and their mothers. A larger sample study is needed to obtain more accurate and reliable results. However, it is very challenging to find large samples in this population, and there is high individual variation between children with VIAD. Second, a longer follow-up period would have been needed to investigate the long-term impacts of the intervention. It is possible that the mothers would have needed an extended intervention to maintain their use of the bodily-tactile strategies and higher EA in interactions with their children with VIAD. Third, given the small sample size, the mothers who participated in this study could have higher EA and more resources in interactions with their children than mothers of children with VIAD in general. Thus, the participant mothers may not represent the group of mothers of children with VIAD in general.

### 4.4 Study implications

Future studies should explore the possible benefits of different types of communicative touch. First, it would be important to learn more about the natural use of communicative touch by studying blind mothers and to learn in more detail how they use touch in interactions with their infants. The findings would be very relevant for developing the use of tactile strategies for parents. Second, it would be important to learn more about the benefits of the tactile strategies used in this study. For instance, the responses of children with VIAD should be evaluated when their parents use different types of anticipatory cues. It would be interesting to find out whether the similarity of the anticipatory cue in relation to the following action (e.g., a movement with legs resembling the first movement of a song) is more informative for the child with VIAD than an anticipatory cue that has only the same location as the following action (e.g., touching the legs before moving the legs in the song). Similarly, the possible benefits of different types of parents’ noticing responses should be investigated. We also need to learn more about manual sign acquisition in children with VIAD through tactile signing. Third, it would be important to determine how early play routines could be used to assess engagement and communication development in children with VIAD. Fourth, future studies could investigate the potential continuum of play routines in the bodily-tactile modality. Lastly, there is a need for studies exploring EA between parents and their children with VIAD at different developmental stages (e.g., preverbal and verbal stages of language development). It would also be important to investigate whether the coder’s knowledge of the clinical group assessed, such as children with VIAD, has an impact on EA assessment.

## 5 Conclusion

The results indicate that the mothers began to use more of the bodily-tactile modality in early social play routines during the bodily-tactile early intervention. The mothers also increased their use of touch in different communicative functions, and they rated the intervention as very useful. Thus, the results indicated that the bodily-tactile early intervention gave mothers more resources for the systematic use of the bodily-tactile modality in interactions with their children with VIAD. Moreover, the children were more emotionally available to their mothers during the intervention and the follow-up compared to the baseline. Overall, the mothers’ increased use of the bodily-tactile modality made interactions more accessible for their children with VIAD, which, in turn, was positively linked to EA. Children’s options for participation depend on accessibility. Thus, children with VIAD may be able to use their full potential for participation only if interactions are made accessible to them through the shareable bodily-tactile modality from the beginning of their lives. This makes both accessibility and affective qualities essential in early interactions between parents and their children with VIAD.

## Data availability statement

The datasets presented in this article are not available because the data that have been used are confidential. Further inquiries should be directed to corresponding author SP, sini.m.peltokorpi@utu.fi.

## Ethics statement

The studies involving humans were approved by HUS Regional Committee on Medical Research Ethics. The studies were conducted in accordance with the local legislation and institutional requirements. Written informed consent for participation in this study was provided by the participants’ legal guardians/next of kin.

## Author contributions

SP: Conceptualization, Data curation, Formal analysis, Funding acquisition, Investigation, Methodology, Visualization, Writing – original draft, Writing – review & editing. SS: Conceptualization, Formal analysis, Investigation, Methodology, Supervision, Validation, Writing – review & editing. AN: Conceptualization, Formal analysis, Investigation, Methodology, Validation, Writing – review & editing. PH: Conceptualization, Investigation, Methodology, Validation, Writing – review & editing. ZB: Methodology, Validation, Writing – review & editing. ML: Conceptualization, Funding acquisition, Investigation, Methodology, Supervision, Validation, Writing – review & editing.
