# Supplementary material for: Bodily-tactile early intervention: a pilot study of the role of maternal touch and emotional availability in interactions between three children with visual impairment and additional disabilities and their mothers
Source: Front Psychol. 2024 Jul 29;15:1439605. doi: 10.3389/fpsyg.2024.1439605 (PMC11325483; doi:10.3389/fpsyg.2024.1439605)
Supplement: Supplementary file 1 [file Data_Sheet_1.pdf]

Appendix

Coding procedure

General instructions

**To be coded**	The bodily-tactile strategies and games used by the mother (see categories below).
**Not to be coded**	Mother's actions that relate to holding or turning the child or lifting him/her up or actions related to adjusting the child's clothes. However, if lifting the child up is part of a game, it is coded.<br>Mother's gestural actions that are not accessible to the child through the bodily-tactile modality (e.g., snapping fingers)<br>Actions related to toys<br>Actions related to the child's seizures or hiccups
**Note**	The previous actions of the child and the mother's speech are important to note when coding the actions.
**Making annotations in Elan**	The coding begins when the mother touches the child and ends when her touching ends. If the mother is already in tactile contact with the child at the starting point (e.g., holding the child's hands), the coding starts when the movement begins and ends when the movement finishes.
**The time aspect**	*Repetitions.* If a repetition of a specific bodily-tactile action (a touch/movement related to noticing, an anticipatory cue, or a tactile sign) occurs within five seconds, the two (or more) similar actions are coded as one action.<br>*Nonconventional games.* If different bodily-tactile actions (e.g., stimulations) occur within five seconds, they are coded as one nonconventional game. Nonconventional and conventional games are always coded separately.<br>NOTE: The play with self-created lyrics and melodies resembling a conventional game is coded as a nonconventional game from the beginning until the end.<br>*Conventional games.* Conventional games are coded from the beginning until the end and also when there are pauses during the game (e.g., pauses between different verses). If the mother plays the same game twice, and there are more than five seconds between the first and second games, the two games are coded separately. However, if the mother needs to finish the game because the child is not well, the coding is paused.<br>NOTE**:** Typically, only one bodily-tactile action is coded at a time. However, it is possible to code two simultaneous bodily-tactile actions if they occur at the same time (e.g., nonconventional games and touches related to noticing can occur simultaneously).<br>NOTE: If a bodily-tactile action is observable and recognizable when appearing partly during the last seconds of the video, it is coded.

CATEGORIES	TO BE CODED:	NOT TO BE CODED:
**Nonconventional games**	✓ Games that include vibrations (e.g., the mother makes a vibration sound with her mouth on the child's foot)<br>✓ The sounds that the mother makes in relation to touching or moving the child (e.g., the mother moves her mouth and makes sounds while holding the child's hand on her mouth)<br>✓ Games that include swinging or bouncing the child in the mother's lap (Note: At least two subsequent actions are needed)<br>✓ Songs with self-created words and bodily-tactile actions (e.g., the mother signs la-la-la-la and moves her child in the rhythm)<br>✓ Repeated tickling (e.g., making "rain" on the child's stomach by tickling)<br>✓ Clapping hands together	❖ Kisses<br>❖ Single actions (e.g., a single movement related to bouncing, swinging or tickling the child)<br>❖ Stretching, massaging, touching, and moving the child without singing or other sound effects<br>❖ Imitations
**Conventional games**	✓ Songs with familiar lyrics and a systematic bodily-tactile frame (e.g., "Wheels on the Bus," "Head, Shoulders, Knees & Toes")<br>✓ Rhymes with a systematic bodily-tactile frame<br>✓ Peek-a-boo	❖ Songs with tactile signs (tactile signs are coded in a different category)
**Anticipatory cues**	✓ Anticipatory touches that have the same location on the child's body as the subsequent action (e.g., the mother touches her child's legs before she grasps them). Typically, these tactile cues occur simultaneously with speech. However, anticipatory cues without speech are coded.<br>✓ Anticipatory movements (e.g., the mother moves the child's legs the same way as in the following game before the game begins)	❖ Touches related to general questions (e.g., the mother touches the child and says, "Was it a nice game?")<br>❖ Touches that have a different location on the child's body than the following action (e.g., the mother touches her child's head and talks about a song that begins with leg movements)
**Noticing**	Subcategories:<br>✓ **I.** Touches related to the mother's responses to the child (e.g., the child moves his/her leg during the play, and the mother responds to it by touching the leg before she continues the play).<br>✓ **II.** Bodily-tactile imitation (e.g., the child rolls on the right side, and the mother imitates the movement with her body when the child's legs are on the mother's shoulders).<br>✓ **III.** Contingent responses to the child's reaching gestures in a form of action (e.g., the mother makes a vibration sound with her mouth on the child's hand after the child has made a reaching gesture toward her).<br>**NOTE:** If the mother moves the child's legs during the game and touches the child's legs after the game, the mother's touch is coded as noticing only in the case of her verbal comments revealing that she had felt the child's leg movements during the game (and her touch is a response to it).	❖ The mother holds the child's hand on her own mouth while she is talking and nodding.<br>❖ The mother kisses the child's hand while she is holding it.
**Tactile signs**	✓ Signs belonging to Finnish Sign Language and self-created signs if they are made on the child's body or by co-active signing.<br>NOTE: The meaning of the sign is coded. It is also coded whether the sign was made during singing or the mother's speech.	❖ The mother touches different parts of the child's body and names those parts.
